# Correlates of domestic waste management and related health outcomes in Sunyani, Ghana: a protocol towards enhancing policy

**DOI:** 10.1186/s12889-017-4537-8

**Published:** 2017-07-03

**Authors:** Henry O. Addo, Elvis J. Dun-Dery, Eugenia Afoakwa, Addai Elizabeth, Amposah Ellen, Mwinfaug Rebecca

**Affiliations:** 1 Department of Population, Family and Reproductive Health, School of Public Health, College of Health Sciences, University of Ghana. Box LG 13, Accra, Ghana; 2 Faculty of Public Health and Allied Sciences, Catholic University College of Ghana, Brong-Ahafo Region, Sunyani, Ghana; 3 Faculty of Public and Allied Science, Catholic University College of Ghana, Brong-Ahafo Region, Sunyani, Ghana

**Keywords:** Waste management, Households, Domestic, Waste generation, Municipal assembly

## Abstract

**Background:**

Domestic waste generation has contributed significantly to hampering national waste management efforts. It poses serious threat to national development and requires proper treatment and management within and outside households. The problem of improper waste management has always been a challenge in Ghana, compelling several national surveys to report on the practice of waste management. However, little is known about how much waste is generated and managed within households and there is a serious dearth of information for national policy and planning. This paper seeks to document the handling and practice of waste management, including collection, storage, transportation and disposal along with the types and amount of waste generated by Households and their related health outcome.

**Methods:**

The study was a descriptive cross-sectional study and used a multi-stage sampling technique to sample 700 households. The study was planned and implemented from January to May 2015. It involved the use of structured questionnaires in the data collection over the period. Factors such as demographic characteristics, amount of waste generated, types of waste bins used within households, waste recycling, cost of disposing waste, and distance to dumpsite were all assessed.

**Results:**

The paper shows that each surveyed household generated 0.002 t of waste per day, of which 29% are both organic and inorganic. Though more than half of the respondents (53.6%) had positive attitude towards waste management, only 29.1% practiced waste management. The study reveals that there is no proper management of domestic waste except in few households that segregate waste. The study identified several elements as determinants of waste management practice. Female respondents were less likely to practice waste management (AOR 0.45; 95% Cl 0.29, 0.79), household size also determined respondents practice (AOR 0.26; Cl 0.09, 0.77). Practice of recycling (AOR 0.03; Cl 0.02, 0.08), distance to dumpsite (AOR 0.45; Cl 0.20, 0.99), were all significant predictors of waste management practice. Cholera which is a hygiene related disease was three times more likely to determine households’ waste management practice (AOR 3.22; Cl 1.33, 7.84).

**Conclusion:**

Considering the low waste management practice among households, there is the need for improved policy and enhanced education on proper waste management practice among households.

## Background

Recent most pressing challenge facing developing countries is the ever accumulating quantity of waste generated without adequate facilities and resources available for management [1, 2]. In effect, the whole world produces over 500 million tons of waste every year [3], with recent studies indicating approximately 41.8 million metric tons of waste generated worldwide [4]. Health and environmental effects, uncertainty regarding regulations and negative perceptions by both waste handlers and generators are important concerns in global healthcare [5]. In recent studies, campaigns against waste infrastructure have emerged in some developed countries, like the US, UK and France, because of the increasing public anxiety about the impacts of industrialism upon the environment and human health [3]. In addition, ample literature from Ghana exists to show that unplanned development, including sheer migration between rural and urban populations, contribute to accelerate the deterioration of urban environmental quality conditions such as waste deposition [6]. Indicating that globally, more than 8000 people die every day from diseases related to poor sanitation and hygiene conditions [7–10]. While other countries focus on recycling and recovery of waste as useful materials and for energy purposes [11], most developing countries including Ghana use the landfilling strategy for waste deposition, which is expected to increase due to countries’ shifting practice from open dumping wastes to landfilling [12]. The practice of poor waste management seems common, as it is reported in India, China and Bangladesh, resulting in health threats to the populations as well as major occupational and environmental risk [13]. Considering the wide range of contaminants from waste and different exposure routes, concern remains about the potential human health risks [14]. Other studies however indicates that the motivation driving hygiene behaviors and improved sanitation are sensory issues of smell, disgust and fear of diseases [15]. From recent studies in Ghana, waste workers are prone to various illnesses and injuries from numerous hazards as a result of waste [16]. About 20% of these wastes pose high risk, either of infection and chemical or radiation exposure [17]. In other countries like India, strict laws makes it mandatory for all waste to be handled and managed without any harm to human health and environment [18]. Ghana’s national waste management guideline have stated that waste should be disposed of at land filled sites and should not be deposited or scattered on the surface of open dumps [19]. Similar literature have however indicated that household generated refuse (solid wastes) if not properly managed may create routes for transmission of microbial agents [20]. Additionally, uncollected solid waste by waste management authorities, as is mostly the case of Ghana, become receptacles of large quantities of human excreta which ends up at refuse dumps and water bodies during torrential rains [20]. This is not limited to waste management authorities. Studies by Kumar and collegues [13]) have reported that the waste management practices even among general populations were not appropriate, hence the need for education and training. In Ghana, even though studies have measured waste management, little is known about the practice of waste management among the general population, especially those generated by households. Hence, this study seeks to investigate the generation and practice of waste management among households in Ghana and their related health outcome.

## Methods

### Study design, area, population, and inclusion criteria

The study used a descriptive cross-sectional survey involving only quantitative data collection methods. The study was done in the Sunyani Municipal in the Brong-Ahafo Region of Ghana. The region is one of the 10 administrative regions in Ghana. The region itself has 17 administrative districts and 2 municipalities of which Sunyani is one. Sunyani is the largest settlement in the Brong Ahafo Region in terms of population and area. The population of the region in 2016, based on the year 2010 Population and Housing Census with a projected growth rate of 2.3% is 2,310,983 [21]. The study involved heads of households with a household composition of at least one, who were residents of Sunyani municipality and were above 18 years of age. For the purpose of this study, a household head is defined as a male or female who is above 18 years of age and who oversees the provision and protection of at least one person with whom he or she lives together with as a unit. This study population was determined by the limited data and research among this group.

### Sampling technique and sample size

The study was cross-sectional and used multistage sampling technique. It was planned and carried out from January to May 2016 because this is a raining season in Ghana and most waste related diseases occur within these months. The survey considered the primary sampling unit to be the household. Simple random sampling technique was used to identify 16 sub districts in the municipality and the survey started in the district that was closer to the capital of the municipality, Sunyani. These included Nkwabeng, South ridge, Estate, New Dormaa, Abesim, Sunyani polytechnic, Penkwase, Area1, Area2, Area3, Area4, Magazine, Zongo, Watchman, Benue Nkwanta and Dr. Berko area. Systematic sampling was used in selecting 700 households by selecting every third house in the community. The tertiary sampling units were a single member per household, preferably the household head, where every third household head was selected and interviewed. This was done until the sample size was reached. Based on the assumption that 50% of households would practice waste management, it was estimated that a sample size of 700 was needed to have a 95% confidence interval with a 5% margin of error. The sample size was adjusted by 5% to make room for the high non-response rate and recording errors, giving a total sample size of 735. A total of 724 individuals responded to the survey questionnaire but only 700 questionnaires were used for the analysis.

### Data collection

The research assistants were made up of undergraduate and graduate students from the social sciences and medicine background. The researchers interacted with the heads of households using a structured interviewer administered questionnaire. The research assistants were extensively trained for two days on the purpose of the survey, the survey procedures and questionnaire. The administration and completion of the questionnaires without any form of coercion, and handling of unresponsive interviewees during the process was also explained. The research assistants were also trained to ensure that the participants are completely informed of their rights prior to obtaining consent and ensuring confidentiality. The survey collected detailed information on respondents at their premises on waste management practice, safety behavior and diseases regarding household waste management, and the cost of managing their household waste. Once a household head was considered eligible, he or she was invited orally to participate in the survey. In total, 724 out of the 735 individuals considered eligible consented.

### Data analysis and statistical procedure

Data was entered into Statistical Package for Service Solutions (SPSS) version 20 data processing software and all entry errors were corrected. This was done after all questionnaires were collated and all differences and errors were rectified. Descriptive analysis was performed to investigate the characteristics of different waste management practices of the study respondents. This generated frequencies and percentages into tables to better describe the results. The second stage of analysis was the uni-variate logistic regression analyses. This was conducted to test the influence of socioeconomic and demographic factors on the households’ waste management practice, their knowledge level of disease causation related to poor waste management, and their safety behavior with regard to waste handling. Considering *p* < 0.05 as significant, all significant variables were put into a multiple logistic regression model to calculate adjusted odds ratio. This was done to control for confounders such as gender, age, household size, cost of waste management, amount of waste generated and authorities responsible for managing waste.

## Results

### Descriptive results

There were 700 study participants consisting of heads of households who were age 18 and above. The results indicate that more than half of the respondents (53%) were male. Those within ages 31 to 40 were 28%, age 41 to 50 were 25% and the younger age group which is below 30 years was only 20%. The rest of the age groups were less than 20%. Majority were Christians (67%) and Muslims (27%). The traditionalist represented only 5%. More than half of the respondents were employed (74%), but only 46% received monthly wages above 500 Ghana cedis ($128), 25% received monthly average of 200 to 500 Ghana cedis ($51 – $128). On marital status, 54% were married and most households’ size was between 4 to 6. Other details are indicated on Table 1.

**Table 1:** Background attributes of respondents (*n* = 700)

Attributes	Frequency	Percent (%)
Gender of respondent		
Male	374	53.4
Female	326	46.6
Age (in years)		
Below 30 years	139	19.9
Age 31–40	198	28.3
Age 41–50	177	25.3
Age 51–60	100	14.3
Above age 60	86	12.3
Religious affiliation		
Christian	471	67.3
Muslim	188	26.9
traditionalist	38	5.4
other religions	3	.4
Current employment status		
Employed	518	74.0
Unemployed	182	26.0
Income level		
High(above_500_gh_cedis)	323	46.1
Average(200-500_gh_cedis)	179	25.6
Low(below_200_gh_cedis)	141	20.1
No income	57	8.1
Marital status		
Married	379	54.1
Not married	321	45.9
Level of education		
Educated	654	93.4
Not educated	46	6.6
Household size		
1–3	260	37.1
4–6	316	45.1
Household size		
1–3	260	37.1
4–6	316	45.1
7+	124	17.7

### Solid waste management practice and related health outcomes

The results indicate that 37% of the respondents generate organic waste, 34% generate inorganic waste and 29% generate both organic and inorganic waste. Among those who generate waste, a little above half of the respondents (58%) used an open container as a receptacle in the household, 23% dumped waste in spaces outside the door while 19% were dumping waste within surroundings of the house. Most respondents (44%) confirmed generating up to 0.002 t of waste, 26% generated 0.003 t and 21% generated .001 t of waste (variable not indicated on table). However, seven in every ten (71%) were not practicing waste management, and another 86% never practiced recycling of waste generated. Among waste management agencies, 66% of individuals managed waste by themselves; the municipal assembly is only responsible for managing 15% of the waste generated (variable not indicated on table). Waste disposal came along with some cost, 73% of the respondents said the cost of disposing waste was moderate and 80% of households were willing to pay for waste disposal. On the frequency of sickness, 44% often reported sick and 70% were of the view that the illness was related to improper waste management. Most respondents reported sick with malaria (45%) (variable not indicated on table), but 54% had a positive attitude towards waste management and 76% dumped refuse at designated dumping sites. 72% confirmed the dump sites were easily accessible. Overall, 63% were doing open dumping at the outskirts of town and another 62% visited the dump site on weekly basis to dispose waste. See Table 2.

**Table 2:** Solid Waste Management Practice and Related Health outcomes

Attributes	Frequency	Percent (%)
Type of waste generated in households		
Organic waste	256	36.6
Inorganic waste	241	34.4
Organic and in organic waste	203	29.0
Type of receptacle used in the house		
In-door open dumping	132	18.9
Out-door open dumping	164	23.4
Open container	404	57.7
Waste management practice		
Yes	204	29.1
No	496	70.9
Practice of recycling		
Yes	95	13.6
No	605	86.4
Rating charges for waste collection		
Expensive	122	17.4
Moderate	508	72.6
No charges	70	10.0
Willingness to pay		
Yes	560	80.0
No	140	20.0
Frequency of people reporting sick in the municipality		
Often	308	44.0
Not at all	392	56.0
Illness related to improper waste management		
Yes	491	70.1
No	209	29.9
People’s attitude towards waste disposal		
Positive	375	53.6
Negative	325	46.4
Place of disposing refuse		
Open bush dumping	167	23.9
Designated refuse dump	533	76.1
If dump site, is it easily accessible		
Yes	505	72.1
No	195	27.9
Type of refuse dump in the Municipality		
Surface dump at the outskirts of town	440	62.9
Community container	153	21.9
No dumping site	107	15.3
Frequency of visiting dump sites		
Daily	204	29.1
Weekly	434	62.0
When container is full	35	5.0
Monthly	27	3.9
Distance to dumpsites		
Too far(above 250 m)	125	17.9
Far(200-250 m)	313	44.7
Close(100-149 m)	262	37.4

### Associations between background attributes of respondents and waste management practice (simple logistic regressions)

A chi-square test was also conducted to ascertain the associations between waste management practice and demographic characteristics of respondents. The results revealed that gender of respondent (*p* < 0.001), respondents’ age (*p* < 0.02), and household size (*p* < 0.002) were statistically significant. On the contrary, religious affiliation, employment status, income level, marital status, and level of education did not have any statistical relationship with waste management. Table 3.

**Table 3:** Associations between background attributes of respondents and Waste management practice

Waste management practice			
Attributes	Yes; n (%)	No; n (%)	*P*-value
Gender of respondent			
Male	133 (35.6)	241 (64.4)	.001
Female	71 (21.8)	255 (78.2)	
Age (in years)			
Below 30 years	444 (31.7)	95 (68.3)	.020
Age 31–40	54 (27.3)	144 (72.7)	
Age 41–50	60 (33.9)	117 (66.1)	
Age 51–60	33 (33)	67 (67)	
Above age 60	13 (15.1)	73 (84.9)	
Religious affiliation			
Christian	131 (27.8)	340 (72.2)	.087
Muslim	54 (28.7)	134 (71.3)	
Traditionalist	18 (47.4)	20 (52.6)	
Other religions	1 (33.3)	2 (66.7)	
Current employment status			
Employed	154 (29.7)	364 (70.3)	.564
Unemployed	50 (27.5)	132 (72.5)	
Income level			
High(above_500_gh_cedis)	81 (25.1)	242 (74.9)	
Average(200-500_gh_cedis)	66 (36.9)	113 (63.1)	
Low(below_200_gh_cedis)	49 (34.8)	92 (65.2)	.141
No income	8 (14)	49 (86)	
Marital status			
Married	103 (27.2)	276 (72.8)	.214
Not married	101 (31.5)	220 (68.5)	
Level of education			
Educated	196 (30)	458 (70)	.070
Not educated	8 (17.4)	38 (82.6)	
Household size			
1–3	92 (35.4)	168 (64.6)	.002
4–6	90 (28.5)	226 (71.5)	
7+	22 (17.7)	102 (82.3)	

### Associations between respondents’ practices, related health outcomes and waste management practice (simple logistic regressions)

Table 4 presents the chi-square test on respondents’ practices and waste management practice. The results indicate that the type of waste generated by households (*p* < 0.001), the type of receptacle used by household (*p* < 0.001), the amount of waste generated (*p* < 0.003) (not indicated on table), and persons responsible for solid waste management (*p* < 0.001) were all significantly related to waste management practice. Similarly, rating charges for waste collection (*p* < 0.005), frequency of people reporting sick (*p* < 0.004), and common diseases occurring in the community (*p* < 0.001), had an influence on waste management practice of respondents. In addition to that, the type of refuse dump in the community (*p* < 0.001), the frequency at which respondents visit the dump site (*p* < 0.001) and the distance to the dump site (*p* < 0.001) also established a relationship with waste management practice. Aside the above, all other variables did not establish any statistical relationship with waste management practice. Table 4.

**Table 4:** Associations between respondents’ practices, related health outcomes and waste management practice

Waste management practice			
Attributes	Yes; n (%)	No; n (%)	*P*-value
Type of waste generated in households			
Organic waste	68 (26.6)	188 (73.4)	.001
Inorganic waste	115 (47.7)	126 (52.3)	
Organic and in organic waste	21 (10.3)	182 (89.7)	
Type of receptacle used in the house			
In-door open dumping	54 (40.9)	78 (59.1)	.001
Out-door open dumping	51 (31.1)	113 (68.9)	
Open container	204 (29.1)	496 (70.9)	
Practice of recycling			
Yes	81 (85.3)	14 (14.7)	.001
No	123 (20.3)	482 (79.7)	
Persons responsible for solid waste			
Individuals	125 (27.1)	336 (72.9)	.001
The municipal assembly	60 (58.3)	43 (41.7)	
Private agencies	4 (16.7)	20 (83.3)	
Not available	15 (13.4)	97 (86.6)	
Rating charges for waste collection			
Expensive	31 (25.4)	91 (74.6)	.005
Moderate	163 (32.1)	345 (67.9)	
No charges	10 (14.3)	60 (85.7)	
Frequency of people reporting sick in the municipality			
Often	107 (34.7)	201 (65.3)	.004
Not at all	97 (24.7)	295 (75.3)	
Common diseases occurring in the community			
Malaria	77 (24.5)	237 (75.5)	.001
Diarrhea	36 (32.4)	75 (67.6)	
Cholera	49 (42.2)	67 (57.8)	
Typhoid	11 (55)	9 (45)	
Other diseases	31 (22.3)	108 (77.7)	
If dump site, is it easily accessible			
Yes	167 (33.1)	338 (66.9)	.001
No	37 (19)	158 (81)	
Type of refuse dump in the Municipality			
Surface dump at the outskirts of town	159 (36.1)	281 (63.9)	.001
Community container	24 (15.7)	129 (84.3)	
No dumping site	21 (19.6)	86 (80.4)	
Frequency of visiting dump sites			
Daily	76 (37.3)	128 (62.7)	.001
Weekly	122 (28.1)	312 (71.9)	
When container is full	4 (11.4)	31 (88.6)	
Monthly	2 (7.4)	25 (92.6)	
Distance to dumpsites			
Too far(above 250 m)	49 (39.2)	76 (60.8)	.001
Far(200-250 m)	108 (34.5)	205 (65.5)	
Close(100-149 m)	47 (17.9)	215 (82.1)	

### Predictors of waste management practice (multiple logistic regressions)

A multiple logistic regression was also conducted with variables that were significant at the simple logistic regression. The model considered variables that were significant at *p*-value of 0.05. The model revealed that the female population were less likely to practice waste management (AOR 0.48, 95% CI: 0.29–0.79) as compared to the male population. Both household size 4 to 6 (AOR 0.26, 95% CI: 0.09–0.77) and household size above seven (AOR 0.35, 95% CI: 0.12–0.98) were all significant but less likely to determine the waste management practice of respondents. On the type of waste generated by households, both generation of organic and inorganic waste were less likely to determine the practice of waste management (AOR 0.27, 95% CI: 0.13–0.58). Recycling is considered an important component of waste management, as a result, those who did not practice recycling were less likely to practice waste management (AOR 0.03, 95% CI: 0.02–08). Private agencies responsible for waste disposal (AOR 0.17, 95% CI: 0.06–0.45), and the usage of community containers (AOR 0.33, 95% CI: 0.14–0.79) were both less likely to determine the outcome. Several diseases were reported to be related to improper waste management practice, but only cholera was more likely to determine the waste management practice of respondents (AOR 3.22, 95% CI: 1.33–7.84). Whether the distance to dumpsite was far (AOR 0.45, 95% CI: 0.20–0.69) or close (AOR 0.39, 95% CI: 0.21–0.99), were also less likely to determine the practice of waste management. Contrary to the above variables, the model failed to establish the other variables as determinants of waste management practice. Other details are presented on Table 5.

**Table 5:** Predictors of waste management practice (multiple logistic regression)

Determinants	Waste management practice		
	Adjusted Odds Ratio	95% CI	
Gender			
Male	Ref		
Female	.479**	.287	.799#*
Age			
Below 30 years	Ref		
Age 31–40	.808*	.284	2.299
Age 41–50	1.175	.426	3.243
Age 51–60	.793	.302	2.080
Above age 60	1.094	.400	2.988
Household Size			
1–3	Ref		
4–6	.262*	.090	.767#*
7+	.345	.122	.977#*
Type of waste generated in households			
Organic waste	Ref		
Inorganic waste	.577**	.270	1.233
Organic and in organic waste	.269	.125	.577#*
Type of receptacle used in the house			
In-door open dumping	Ref		
Out-door open dumping	1.179**	.599	2.318
Open container	1.338	.710	2.522
Amount of waste generated in households			
0.001 t	Ref		
0.002 t	1.326**	.349	5.031
0.003 t	.767	.208	2.821
0.004 t	1.529	.390	6.000
Practice of recycling			
Yes	Ref		
No	.030**	.015	.077#*
Persons responsible for solid waste			
Individuals	Ref		
The municipal assembly	.539**	.224	1.296
Private agencies	.166	.062	.449#*
Not available	1.413	.211	9.454
Rating charges for waste collection			
Expensive	Ref		
Moderate	1.508*	.452	5.033
No charges	1.317	.410	4.230
Frequency of people reporting sick in the municipality			
Often	Ref		
Not at all	.700*	.411	1.193
Common diseases occurring in the community			
Malaria	Ref		
Diarrhoea	1.788**	.843	3.790
Cholera	3.222	1.325	7.835#*
Typhoid	1.563	.661	3.694
Other diseases	.209	.044	.988#*
Type of refuse dump in the Municipality			
Surface dump at the outskirts of town	Ref		
Community container	.328**	.137	.787#*
No dumping site	.471	.163	1.358
Frequency of visiting dump sites			
Daily	Ref		
Weekly	.757**	.075	7.599
When container is full	.714	.076	6.736
Monthly	.596	.050	7.112
Distance to dumpsites			
Too far(above 250 m)	Ref		
Far(200-250 m)	.449**	.202	.995#*
Close(100-149 m)	.383	.214	.686#*

95% CI: Confidence interval, * *p* < 0.05, ** *p* < 0.001, ref: Reference, #*significant CI

## Discussion

The analysis of results suggest that more than half of the respondents were male as compared to findings by Yuan and Yabe [22] in Beijing, where majority of their respondents were the female population. This high percentage may be because the study was conducted at a time that most female would have been out of the house on farm or feminine duties. The current study reported a majority age group between 31 and 40. In similar studies in Ghana, Yoada and colleauges, [23]) indicated that majority of their study respondents were within this age group and more than half were married, a finding also supporting that of this study.

Even though several literature have cited inorganic components of waste to be quite problematic because of their non-biodegradable nature and lasting effect on the environment [24], a greater proportion of respondents still generate inorganic waste. Indicating that both studies reveal the effects of inorganic waste on both human life and the environment. Contrary to this, more than half of waste generated by respondents in Tehran was biodegradable [25], just a little of such was cited by this study. Regional variations in consumption patterns, production needs and economic policies may sometimes account for such difference in waste generation, but such patterns is an indication of less negative impact on the environment and human health, as most waste generated are easily managed naturally. Nagarajan et al., [26]), in their study in India stated that about 75 t of garbage are generated daily and the daily per capita generation of solid waste ranges from 100 g to 500 g. This however, is far higher than the findings of this study, as this study cited only 0.002 t of waste generated by households. In assessing the level of waste management practice, respondents were asked three main questions which include whether they practiced waste segregation, if they used closed waste bins and if they dispose off waste at designated places. Their responses were then added to give the percentage of respondents practicing waste management.

Data from this study indicate that respondents’ age was a determinant of their waste management practice. Contrary to this, findings by Mamady [27]), suggest that age was not a determinig factor for the practice of waste management. In both this study and the above cited study [27], the gender of a respondent influenced his or her practice of waste management. An earlier study [28], have indicated that there exist a relationship between availability of waste experts and waste management practice, a finding that concur with findings from this current study. Even though both studies have ranked the attitude of respondents to waste management as positive, the current study did not establish any relationship between positive attitude and the practice of waste management. Like this study, earlier studies in Ghana [29] have also established the link between improper waste management practice and disease occurrence and both studies further cited cholera as a common disease resulting from improper waste management. Considering that cholera outbreaks are as a results of poor sanitation, open space refuse dumps within communities can predispose inhabitants to cholera infection, a practice that has been indicated by the current study but was not a determinant of waste management practice. In the current study, distance from respondents’ residence to the waste dumpsite was a predictor of their practice of waste management. This is however contrary to results by Mahmoudkhani and colleaggues [12]) in similar studies done in Iran. This could explain why only 62% of respondents visited the dumpsite on weekly bases and the practice of waste management was as low as 29%.

Available data from a multivariate analysis indicate that sex was an independent predictor of the practice of waste management and the female population was less likely to practice waste management as compared to the male population. In contrary findings, previous literature identified the female population as more likely to influence the practice of waste management [27]. This should be explained with the fact that the female population is mostly house keepers and tend to dominate household sanitation issues. Further studies that seems to agree with findings of this studies are findings by UETA, [30]), where increase in household size tend to reduce the quantity of household waste and the practice of waste management. In this study, household sizes of 4 to 6 and above 7 were less likely to engage in the practice of waste management as compared to household size below 4 people. Conversely, previous studies by Osbjer et al. [31]), found that household practice of waste management is associated with a higher number of people in the households, which could possibly be explained by the need to handle waste generated by larger populations within the household. In considering other variables, respondents of this study did not practice waste management as a result of their household income level; however, the opposite is said of other studies done in Kenya where income significantly influenced the method of waste disposal among households. As compared to individual and local government waste management contractors, respondents acknowledge that the private waste management authorities predominantly influenced their practice of waste management, though the cost of contracting them did not influence their practice. This, however, did not conform with earlier findings elsewhere in Ghana, where most respondents complained of irregular patterns in waste collection and the high cost of contracting private waste collectors [23]. Evidently, other literature further state that respondents with higher income commonly preferred accredited private sector as waste collectors [27]. It is certain that the practice of contracting private waste management agencies and their related cost will have consequences on global efforts to achieving the millennium development goal seven, which is more critical on ensuring environmental sustainability.

Surprisingly, while diseases like malaria were commonly reported among respondents, contrary to asthma reported by Maina & Muriuki, [32]) in Kenya, the frequency of occurrence and types diseases commonly reported were not predictors of waste management practice. Throughout literature, the importance of waste bins and containers for the management of waste have been emphasized [24, 32–34], and other writers suggest they offer a more cost- effective waste management service, considering that they improve household waste separation and reduces the amount of waste in landfills [23]. In line with this study, the presence of a community container indicated an influence on the practice of waste management. While Tadesse & Kumie, [5]), found that even though containers were present, most had no lids and waste were usually stored for a period of more than 24 h – an indication of poor waste management that suggest a health hazard for both human and environmental health. Like previous literature [27], distance to permitted dumpsites for respondents of this study, was a reason to practice waste management, even though this may also lead to other health complications and mortalities from certain cancers [35]. This study is not without limitations. A major limitation associated with this study is that, only households were included in the study, excluding the views and practices of waste management authorities and office-based establishments. The study was also limited to only Sunyani Municipality, which is considered a small portion of the regional population. It will therefore be inappropriate to generalize the findings of the study. Authors also consider recall bias as one limitation of the study, given that respondents had experienced the practice and it would have been difficult for respondents to remember all experiences during the period under study.

## Conclusions

Even though respondents were knowledgeable about waste management, its practice was low. Most respondents still did open dumping at the outskirts of town and a greater proportion still used open containers as waste bins. Respondents also had fair knowledge about improper waste management related diseases but this knowledge did not translate into practice. The greatest challenge mentioned by respondents was however the cost of paying for waste disposal, especially when contracting a private waste management agency. In most households, both organic and inorganic waste were generated, explaining the reasons the practice of recycling predicted waste management practice, even though majority did not adhere to this practice.
